# Effect of the Source of Zinc on the Tissue Accumulation of Zinc and Jejunal Mucosal Zinc Transporter Expression in Holstein Dairy Calves

**DOI:** 10.3390/ani10081246

**Published:** 2020-07-22

**Authors:** Fengtao Ma, Yeqianli Wo, Hongyang Li, Meinan Chang, Jingya Wei, Shengguo Zhao, Peng Sun

**Affiliations:** State Key Laboratory of Animal Nutrition, Institute of Animal Science, Chinese Academy of Agricultural Sciences, Beijing 100193, China; fengtaoma@163.com (F.M.); wyql0427@163.com (Y.W.); 15840076108@163.com (H.L.); meinan0616@126.com (M.C.); 18702716486@163.com (J.W.); zhaoshengguo@caas.cn (S.Z.)

**Keywords:** zinc-methionine, zinc oxide, diarrhea, zinc transporters, dairy calf

## Abstract

**Simple Summary:**

Diarrhea is the main cause of death in newborn calves and is associated with antibiotic use and economic loss for dairy farms. In this study, we evaluated the effects of different sources of the mineral zinc (zinc oxide (ZnO) and zinc methionine (Zn-Met)) on the growth, incidence of diarrhea, tissue zinc accumulation, gene expression of jejunal zinc transporters and serum concentrations of zinc-dependent proteins in newborn Holstein dairy calves. We found that Zn-Met supplementation promoted growth and reduced diarrhea from the second week after birth. It also increased the levels of zinc in the serum and liver, the level of the transporter protein ZIP4 in the jejunal mucosa, as well as the serum alkaline phosphatase and metallothionein concentrations compared to the control group. ZnO supplementation had similar but less marked effects to Zn-Met supplementation. These results suggest that Zn-Met supplementation may be an alternative to antibiotics for the treatment of newborn calf diarrhea.

**Abstract:**

Zinc is considered to be an anti-diarrheal agent, and it may therefore reduce the incidence of diarrhea in young calves. In the present study, we aimed to compare the effect of zinc source on growth performance, the incidence of diarrhea, tissue zinc accumulation, the expression of zinc transporters, and the serum concentrations of zinc-dependent proteins in neonatal Holstein dairy calves. Eighteen male newborn Holstein dairy calves were fed milk and starter diet supplemented with or without 80 mg zinc/d in the form of Zn-Met or ZnO for 14 days, and were then euthanized. Zn-Met supplementation improved average daily gain and feed efficiency, and reduced the incidence of diarrhea, compared with control calves (*p* < 0.05). It also increased the serum and hepatic zinc concentrations and the mRNA expression of the ZIP4 transporter in the jejunal mucosa of the calves (*p* < 0.05). In addition, the serum alkaline phosphatase activity and metallothionein concentration were higher in Zn-Met-treated calves than in control calves (*p* < 0.05). ZnO supplementation had similar effects, but these did not reach significance. Thus, Zn-Met supplementation is an effective means of increasing tissue zinc accumulation and jejunal zinc absorption, and can be used as an anti-diarrheal strategy in neonatal calves.

## 1. Introduction

Diarrhea is the primary cause of death in calves, especially during the first two weeks after birth, and this results in the use of antimicrobials and economic loss for dairy farms [1]. To reduce antimicrobial use on dairy farms, the identification of effective anti-diarrheal agents is of great importance.

Zinc is a trace element that is essential for all living organisms [2]. It is an important component of many enzymes that are involved in diverse physiological and cellular functions, including in various immunological, endocrine, neuronal, and reproductive processes [3]. In recent years, zinc has also been used as an anti-diarrheal agent to ameliorate diarrhea in infants and children [4]. In animal production, high doses of zinc oxide (ZnO, 2000–4000 mg/kg) have been used to reduce the incidence of diarrhea and promote growth in young animals, but this is associated with the fecal loss of large quantities of zinc [5]. Because of the environmental impact, supplementation with high doses of ZnO was banned by the Ministry of Agriculture and Rural Affairs of China in 2017.

We previously evaluated the optimal dose of zinc for neonatal calves and showed that 80 mg zinc, in the form of ZnO, has the potential to alleviate newborn calf diarrhea [6]. In a subsequent study, we compared the effects of the same dose of zinc, provided in organic or inorganic form, on the growth and incidence of diarrhea in dairy calves, and showed that a low dose of zinc methionine (Zn-Met) or ZnO can ameliorate diarrhea in young calves, which is not the result of effects on rectal microbial composition or diversity [7].

In the present study, we investigated the effects of the same dose of zinc provided in two different forms on tissue zinc accumulation and zinc absorption in dairy calves during the first two weeks after birth by assessing their growth performance, incidence of diarrhea, tissue zinc content, jejunal mucosal zinc transporter expression, and serum concentrations of zinc-dependent proteins.

## 2. Materials and Methods

This study was conducted at Beijing Sino Farm (Beijing, China). All procedures were approved by the Institute of Animal Science, Chinese Academy of Agricultural Sciences (Beijing, China). All the animals used in the present study were raised according to standards established by the Institute of Animal Science, Chinese Academy of Agricultural Sciences (Beijing, China).

### 2.1. Animals, Diets, and Experimental Design

Eighteen healthy, newborn male Holstein dairy calves with similar body weight (40.74 ± 0.63 kg) were randomly allocated to one of three treatment groups (*n* = 6 each). The groups were as follows: (1) control group (CON, no supplemental ZnO or Zn-Met provided); (2) Zn-Met group (consuming 455 mg Zn-Met per day, equivalent to 80 mg zinc); and (3) ZnO group (consuming 103 mg ZnO per day, equivalent to 80 mg zinc). The supplemented zinc level was based on previous publications [6,8,9]. The trial lasted for 14 days. Calves were removed from their dams immediately after birth and housed in individual pens (1.8 × 1.4 × 1.2 m) that were bedded with straw and enclosed with iron fences. All the calves consumed 4 L colostrum within 1 h of birth and were then fed the same raw milk. From days 2–3, the calves were fed 2 L raw milk twice a day (at 8:30 and 16:00 h) from a bottle. On days 4–14, a total of 8 L milk was fed daily using a bucket. Zn-Met or ZnO were mixed with 200 mL colostrum or milk and provided to the calves on days 1–14, as described in our previous study [6]. Then, more colostrum or milk were fed to the calves. The commercial pelleted starter diet (Beijing Sanyuan Seed Technology Co., Ltd., Beijing, China) was fed to the calves from day 4. Zinc in the starter diet is provided in the form of zinc sulfate. The nutritional composition of the milk and starter diet are shown in Table 1. The calves were provided ad libitum with water and starter diet.

### 2.2. Sampling and Analysis

On days 1, 7, and 15, before the feed was provided, body weight, height, length, and heart girth were measured to calculate average daily gain (ADG) and mean gains in height, body length, and heart girth. Total feed intake, including milk and starter intake, was also measured throughout the experimental period to calculate average daily feed intake (ADFI, calculated on a dry matter basis) and feed efficiency.

Fecal consistency was scored daily on a 4-point scale as previously described [10]. Diarrhea was defined as a fecal score of 3 or 4 on 2 successive days. The number of calves that had diarrhea and the number of days they had diarrhea during the study were recorded. The incidence of diarrhea was calculated as the number of calves with diarrhea × the number of days with diarrhea/(number of calves in each group × the length of the trial in days) × 100%.

A blood sample was obtained from the jugular vein using vacutainer tubes (BD Biosciences, San Jose, CA) before the morning feed (08:30 h) on day 15. Serum was separated by centrifugation at 3000× g for 15 min at 4 °C and stored at −20 °C. Thereafter, all the calves were euthanized. Liver samples were dissected and washed with ice-cold PBS (pH 7.4) to remove blood and snap-frozen in liquid nitrogen for later analysis. Subsequently, 7–10 cm lengths of jejunum were dissected and rinsed with saline, and then the jejunal mucosa was exposed by a longitudinal incision and scraped using sterile glass slides, and the material obtained was stored at −80°C for subsequent measurement of zinc transporter expression.

Serum alkaline phosphatase (ALP) and superoxide dismutase (SOD) activities, and concentrations of metallothioneins (MTs), growth hormone (GH), and insulin-like growth factor-I (IGF-I), were determined using commercial assay kits (Nanjing Jian Cheng Bioengineering Institute, Nanjing, China), according to the manufacturer’s instructions. The zinc concentrations in the water and milk, the concentrations of calcium, phosphorus, zinc, iron and copper in the starter diet, and the serum and hepatic concentrations of copper and iron were measured by inductively-coupled plasma optical emission spectroscopy, as described in the Chinese National Standards (GB 5009.268, China, 2016). No zinc was detectable in the water. Milk and starter diet contained 4.08 and 175 mg/kg Zn, respectively.

The mRNA expression of the zinc transporters ZnT1, ZnT2, ZnT5, and ZRT-IRT-like protein 4 (ZIP4) was determined using real-time (RT) PCR. RNA was extracted from the jejunal mucosa using Trizol reagent (Invitrogen, Shanghai, China), and then complementary DNA (cDNA) was synthesized using a PrimeScript RT reagent kit and a gDNA Eraser kit (Perfect Real Time; RR047A; Takara, Dalian, China). The sequences of the PCR primers used are shown in Table 2. The PCR reaction buffer was TB Green^TM^ Premix Ex Taq^TM^ II (Tli RNaseH Plus, RR820A; Takara, Dalian, China), and the reaction mixture (25 μL) contained 12.5 μL TB Green Premix Ex Taq (Tli RNaseH Plus) (2×), 2.0 μL cDNA, 1 μL forward primer, 1 μL reverse primer, and 8.5 μL doubled-distilled water. Real-time PCR was conducted using an initial step at 95 °C for 30 s, followed by 40 cycles of 5 s at 95 °C and 30 s at 60 °C on a CFX96 PCR cycler (Bio-Rad, Hercules, CA). Each sample was amplified in triplicate. The mean Ct was calculated for each target gene and *Actb*, and the ΔCt (Ct_target gene_ − Ct*_Actb_*) was determined, where Ct is the number of cycles required to reach the detection threshold. The relative expression of each target (expressed as a fold difference from CON) was calculated using the 2^−ΔΔCt^ method.

### 2.3. Statistical Analysis

The incidence data were analyzed using logistic regression (GENMOD procedure) and a binomial error distribution, with diet as a fixed effect. The link function was a logit transformation. The other data were analyzed by one-way ANOVA using the mixed procedure of SAS (version 9.4; SAS Institute Inc., Cary, NC, USA). Multiple comparisons were performed using Tukey’s multiple range test. Statistical significance was accepted when *p* ≤ 0.05, and trends were reported when *p* < 0.10.

## 3. Results

### 3.1. Growth Performance and Incidence of Diarrhea

The effects of zinc source on the growth performance and incidence of diarrhea in postnatal dairy calves during days 1–7, 8–14, and 1–14 are displayed in Table 3. There were no differences in the ADG, ADFI, and feed efficiency of the calves between the three treatment groups during days 1–7. However, the ADG was significantly higher in the calves receiving Zn-Met supplementation during days 8–14 and across the entire study period than those in the CON group (*p* < 0.05). The feed efficiency was better in the Zn-Met group compared to the CON group during days 8–14 and 1–14 (*p* < 0.05). There were no differences in the height, length, or heart girth gains of the dairy calves in the three treatment groups.

As shown in Table 3, supplementation with Zn-Met reduced the incidence of diarrhea during days 8–14 and across the entire study period versus the CON group (*p* < 0.05). ZnO had a similar effect to Zn-Met, but this did not achieve significance.

### 3.2. Serum and Hepatic Micronutrient Concentrations

Table 4 shows the micronutrient concentrations in the serum and liver of dairy calves. Compared with the CON group, calves in the Zn-Met group had higher serum and hepatic zinc concentrations (*p* < 0.05). Supplementation with ZnO tended to increase serum copper concentration compared with the CON group (*p* = 0.09). However, the addition of zinc did not affect the serum or hepatic copper or iron concentrations of the calves.

### 3.3. Serum Zinc-Dependent Protein Concentrations

The serum concentrations of zinc-dependent proteins in the three groups are shown in Table 5. The serum ALP activity and MT concentrations were higher in the Zn-Met group than in the CON group (*p* < 0.05). There were no significant differences in SOD activity, GH or IGF-Ι concentration between the three groups. Neither ZnO nor Zn-Met supplementation affected the serum concentrations of zinc-dependent proteins in the dairy calves.

### 3.4. RNA Expression of Jejunal Mucosal Zinc Transporters

**Figure 1:**
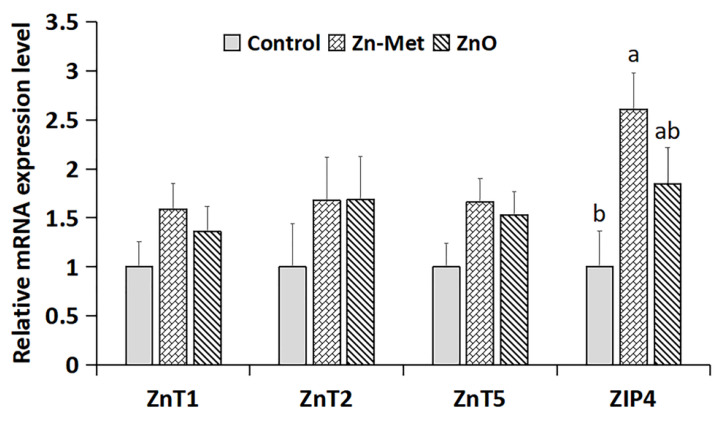
The mRNA expression of zinc transporters (ZnT1, ZnT2, ZnT5, and ZIP4) in the jejunum of Holstein dairy calves. Treatments: CON group, fed only milk and starter diet; Zn-Met (zinc-methionine) group, fed milk and starter diet containing 455 mg Zn-Met (equivalent to 80 mg zinc); ZnO (zinc oxide) group, fed milk and starter diet containing 103 mg zinc oxide (equivalent to 80 mg zinc). Values are means, with their standard errors represented by vertical bars (*n* = 6). Mean values with different superscripts are significantly different (*p* < 0.05).

As shown in Figure 1, there were no differences in the mRNA expression of ZnT1, ZnT2, and ZnT5 in the jejunal mucosa of the dairy calves in the three groups. However, ZIP4 mRNA expression in the jejunal mucosa of the calves supplemented with Zn-Met was higher than in that of the CON calves (*p* < 0.05), but there was no difference between the ZnO and CON groups.

## 4. Discussion

In the present study, we have shown that supplementation with a low dose of Zn-Met increases the growth performance of dairy calves by increasing ADG and feed efficiency during the first two weeks of life. Although ZnO supplementation has similar effects to Zn-Met, there were no significant differences between the ZnO and CON groups. These findings are consistent with those of many previous studies that demonstrated that organic forms of zinc have greater growth-promoting effects than inorganic forms [11,12,13]. Furthermore, the incidence of diarrhea was significantly lower in the Zn-Met group than in the CON group from the second week after birth, as reported previously [14,15]. Considering that the first two weeks of life is when the prevalence of diarrhea peaks in calves, we have recommended Zn-Met supplementation of the diet for dairy calves during early life because this could reduce the incidence of diarrhea [7]. Feldmann et al. [8] also demonstrated that calves whose feed was supplemented with Zn-Met had a 14.7% lower risk of diarrhea than non-supplemented calves.

Dietary zinc intake is essential because zinc cannot be stored in the body permanently [16]. Ingested zinc is absorbed in the intestine and is transported in the portal circulation to the liver [17]. Therefore, the liver is particularly affected by the level of dietary zinc and stores zinc for short periods [18]. Therefore, serum and hepatic zinc concentrations would be expected to increase when dairy calves consume diets supplemented with zinc. Deng et al. [16] proposed that zinc supplementation is an effective way of increasing serum zinc accumulation. Furthermore, Bao et al. [19] reported an increase in the hepatic zinc concentration as the level of dietary supplementation increased, and Wright and Spears [20] showed increases in both the plasma and liver zinc concentrations of Holstein calves following dietary zinc supplementation. Organic zinc has been shown to be more effective than inorganic forms [18]. Shaeffer et al. [21] demonstrated that plasma zinc concentration is higher in steers fed organic zinc than in those fed inorganic zinc, and Kincaid et al. [22] also showed higher serum and liver zinc concentrations in calves administered 300 mg zinc in a mixture of Zn-Met and zinc lysine than in those administered ZnO. In addition, Holstein calves fed an organic source of zinc had higher concentrations of zinc in the plasma and liver than those fed inorganic zinc [20]. Consistent with these findings, we have shown that the serum and hepatic zinc concentrations of dairy calves in the Zn-Met group were higher than those in the CON group, but no differences were observed between the ZnO and CON groups, which implies that Zn-Met has higher bioavailability.

For decades, high doses of zinc have been used for their anti-diarrheal and growth-promoting effects in young animals [2]. However, the use of excessive amounts of zinc wastes resources and may lead to interactions with other metal ions [9,16,23]. It is known that if zinc is ingested in large amounts, it may compete with copper and iron for absorption [24]. Although supplementation with zinc in the form of Zn-Met increased serum and hepatic zinc concentrations, it did not affect iron or copper concentrations in the dairy calves, which implies that dietary zinc supplementation does not interfere with the absorption of iron or copper under the conditions in which the present study was conducted. This may be attributable to the relatively low dose of zinc provided by the amount of Zn-Met or ZnO added to the diet, as shown in our previous study [6]. Similar results were also obtained by Jia et al. [25], who found no effect of zinc supplementation on the serum concentrations of copper and iron in Cashmere goats.

One of the key physiological roles of zinc is as a critical component of various metalloenzymes, which participate in almost every metabolic activity in the body [23,26,27]. ALP is a zinc-containing metalloenzyme, and its activity is used as an indicator of zinc nutritional status [28,29]. Spears [30] found that the plasma ALP activity at 28 and 42 days of age was higher in lambs consuming diets that were supplemented with zinc. MTs are low-molecular weight, cysteine-rich proteins [31] that bind zinc with high affinity, and are involved in zinc absorption, transportation and storage [31]. Dietary supplementation with zinc induces MT synthesis [32], and Swinkels et al. [33] demonstrated that the concentrations of MT in body fluids and tissues are the best markers of zinc availability from diet and mineral sources. Furthermore, Wright and Spears [20] found that zinc supplementation increases hepatic MT concentration in calves. In agreement with these previous findings, serum ALP activity and MT concentration were higher in dairy calves that received Zn-Met supplementation than in CON calves in the present study.

The absorption of zinc in the jejunum is mediated by specific zinc transporters that belong to two major gene families: the solute carriers 30 (SLC30; ZnT) and the solute carriers 39 (SLC39; ZIP). These two types of transporters together regulate and maintain systemic and cellular zinc homeostasis in animals [34], which is threatened when dietary zinc supply is poor or excessive [35,36]. ZnT transporters control zinc efflux from the cytosol into the extracellular space [34,37]. ZIP transporters regulate zinc influx from the extracellular space and intracellular compartments into the cytosol [38,39]. In the present study, Zn-Met supplementation increased the expression of ZIP4 in the jejunal mucosa of dairy calves, suggesting that Zn-Met may promote zinc absorption. This finding is consistent with the increases in serum and hepatic zinc concentrations of the Zn-Met-treated dairy calves. By contrast, Zn-Met supplementation did not affect the mRNA expression of ZnT1, ZnT2, or ZnT5 in the jejunal mucosa of the dairy calves. However, overall, our findings suggest that dietary supplementation with 80 mg zinc might be appropriate for the needs of postnatal dairy calves.

## 5. Conclusions

In the present study, we have shown that supplementation with a low dose of zinc in the form of Zn-Met reduces the incidence of diarrhea and improves the growth of dairy calves from their second week of life. Furthermore, Zn-Met has positive effects on tissue zinc accumulation, the expression of jejunal mucosal zinc transporters, and the serum concentrations of zinc-dependent proteins in postnatal Holstein dairy calves during their first two weeks of life. Supplementation with the same dose of zinc in the form of ZnO had similar effects to those of Zn-Met, but these did not reach significance. Thus, zinc supplementation is effective in ameliorating diarrhea and improving tissue zinc accumulation and jejunal zinc absorption and transport. In addition, organic zinc has higher bioavailability than inorganic zinc. Our findings suggest that Zn-Met could be used as an anti-diarrheal agent in place of antibacterials in the rearing of young calves.

## Figures and Tables

**Table 1:** Nutrient composition of the milk and starter diet (%, unless noted) ^1^.

Item	%
Milk composition	
Density, kg/L	1.02
Protein	3.85
Fat	4.21
Total solids	12.9
Dry matter	12.0
Lactose	4.68
Nutrient composition of the starter diet (Dry matter basis)	
Dry matter	89.3
Crude protein	19.8
Ether extract	2.68
Ash	6.52
Acid detergent fiber	8.03
Neutral detergent fiber	17.6
Ca	0.89
P	0.46
Cu, mg/kg	50.7
Fe, mg/kg	271
Zn, mg/kg	175

^1^ Analyzed value.

**Table 2:** Sequences of primers used to measure gene expression in the jejunal mucosa of calves by quantitative PCR.

Target	Accession Number	Primer Sequences	PCR Product Size(bp)
β-actin	NM_173979.3	F: 5’ ATCCTGCGGCATTCACGAA 3’	154
		R: 3’ TGCCAGGGCAGTGATCTCTT 5’	
ZnT1	NM_001205893.2	F: 5’ GCAACTTGCTGGAAGCAGAA 3’	135
		R: 3’ TCAGGCTGAATGGTGGTAGC 5’	
ZnT2	NM_001191496.1	F: 5’ TCTCCCTGTGGGTGTCTTCC 3’	137
		R: 3’ TTCTGCCGCCAAGTACACC 5’	
ZnT5	NM_001192174.2	F: 5’ GGCTAAAATGGCTGAACACCC 3’	130
		R:3’ ACACAAAGCCAGTACTAGCAACA 5’	
ZIP4 ^1^	NM_001046067.1	F: 5’ CTCTTGCTGCCCCTGGAC 3’	157
		R: 3’ CCACCAGATCTGCGCGAG 5’	

^1^ ZIP4, ZRT-IRT-like protein 4, which is the solute carrier family 39 member 4 (SLC39A4).

**Table 3:** Effects of zinc methionine (Zn-Met) and ZnO on the growth performance and incidence of diarrhea in Holstein dairy calves.

Item	Treatment ^1^			SEM ^2^	*p* Value
	Control	Zn-Met	ZnO		
Days 1 to 7 after Birth					
Average daily gain, g/d Mean height gain, cm	398	485	443	32.9	0.208
	1.92	2.58	2.42	0.51	0.641
Mean body length gain, cm	2.67	1.83	2.67	0.96	0.782
Mean heart girth gain, cm	2.00	3.00	2.73	0.66	0.551
Average daily milk intake, g DM/d	982	982	983	0.80	0.550
Average daily starter intake, g DM/d	11.9	12.9	9.04	3.33	0.700
Average daily total feed intake, g DM/d	994	995	992	2.83	0.774
Total zinc intake, mg/d	6.09 ^b^	86.3 ^a^	85.6^a^	0.58	<0.001
Feed efficiency, g DMI ^3^/g gain	2.56	2.13	2.30	0.18	0.255
Incidence of diarrhea, %	19.0	14.3	11.9	--	0.353
Days 8 to 14 after birth					
Average daily gain, g/d	407^b^	513^a^	433^b^	18.5	0.003
Mean height gain, cm	1.50	2.00	1.92	0.76	0.884
Mean body length gain, cm	2.50	3.75	3.17	0.71	0.477
Mean heart girth gain, cm	2.67	1.96	2.42	0.91	0.856
Average daily milk intake, g DM/d	983	983	983	0.99	0.937
Average daily starter intake, g DM/d	33.0	33.1	37.7	11.9	0.950
Average daily total feed intake, kg DM/d	1.02	1.02	1.02	0.01	0.931
Total zinc intake, mg/d	9.78 ^b^	89.8 ^a^	90.6 ^a^	2.07	<0.001
Feed efficiency, g DMI/g gain	2.54 ^a^	1.99 ^b^	2.37 ^a^	0.11	0.006
Incidence of diarrhea, %	31.0	16.7	23.8	--	0.036
Days 1 to 14 after birth					
Initial body weight, kg	40.5	42.0	39.8	1.08	0.349
Final body weight, kg	46.1	49.0	45.9	1.09	0.115
Average daily gain, g/d	402 ^b^	499 ^a^	438 ^ab^	20.4	0.014
Mean height gain, cm	3.42	4.58	4.33	0.98	0.683
Mean body length gain, cm	5.17	5.58	5.83	0.97	0.888
Mean heart girth gain, cm	4.67	4.96	5.15	0.62	0.860
Average daily milk intake, g DM/d	982	983	983	0.59	0.582
Average daily starter intake, g DM/d	25.4	25.8	31.0	7.22	0.829
Average daily total feed intake, kg DM/d	1.01	1.01	1.01	0.01	0.759
Total zinc intake, mg/d	7.29 ^b^	87.4 ^a^	88.2 ^a^	0.93	<0.001
Feed efficiency, g DMI/g gain	2.54 ^a^	2.04 ^b^	2.33 ^ab^	0.11	0.016
Incidence of diarrhea, %	50.0	31.0	35.7	--	0.033

^a,b^ Mean values in the same row with different superscripts are significantly different (*p* < 0.05). ^1^ Treatment: CON (control), fed only milk and starter diet; Zn-Met group, fed milk and starter diet containing 455 mg Zn-Met (equivalent to 80 mg zinc); ZnO group, fed milk and starter diet containing 103 mg zinc oxide (equivalent to 80 mg zinc). ^2^ SEM, standard error of the mean. ^3^ DMI, dry matter intake.

**Table 4:** Effects of Zn-Met and ZnO on the serum and tissue micronutrient concentrations of Holstein dairy calves.

Item	Treatment ^1^			SEM	*p* Value
	Control	Zn-Met	ZnO		
Serum micronutrient concentration (mg/kg)					
Zinc	2.06 ^b^	3.34 ^a^	2.82 ^ab^	0.32	0.037
Iron	4.26	4.92	4.48	0.62	0.744
Copper	0.77	0.87	1.22	0.14	0.090
Hepatic micronutrient concentration (mg/kg) ^2^					
Zinc	75.5 ^b^	122^a^	117 ^ab^	11.7	0.025
Iron	34.5	35.4	35.4	3.83	0.982
Copper	83.5	84.9	83.5	10.2	0.994

^a,b^ Mean values in the same row with different superscripts are significantly different (*p* < 0.05). ^1^ Treatment: CON (control), fed only milk and starter diet; Zn-Met group, fed milk and starter diet containing 455 mg Zn-Met (equivalent to 80 mg zinc); ZnO group, fed milk and starter diet containing 103 mg zinc oxide (equivalent to 80 mg zinc). ^2^ The results are fresh weight basis.

**Table 5:** Effects of Zn-Met and ZnO on the serum zinc-dependent protein concentrations of Holstein dairy calves.

Item	Treatment ^1^			SEM	*p* Value
	Control	Zn-Met	ZnO		
Alkaline phosphatase, ng/mL Metallothionein, pg/mL	1.55 ^b^ 768 ^b^	1.74 ^a^	1.73 ^a^	0.05	0.034
		906 ^a^	874 ^ab^	36.0	0.040
Superoxide dismutase, U/mL	76.9	79.5	78.8	3.15	0.835
Growth hormone, pg/mL	3.91	4.21	3.94	0.19	0.480
Insulin-like growth factor-Ι, ng/mL	11.7	11.1	10.9	0.85	0.810

^a,b^ Mean values in the same row with different superscripts are significantly different (*p* < 0.05). ^1^ Treatment: CON (control), fed only milk and starter diet; Zn-Met group, fed milk and starter diet containing 455 mg Zn-Met (equivalent to 80 mg zinc); ZnO group, fed milk and starter diet containing 103 mg zinc oxide (equivalent to 80 mg zinc).
